# Primary Pulmonary Marginal Zone Lymphoma Presenting With Asymptomatic Lung Consolidation

**DOI:** 10.1155/crpu/7871010

**Published:** 2026-02-18

**Authors:** Mohammed Al Shuraiqi, B. Jayakrishnan, Mohammed Al Harrasi, Zahra Al Hajri, Radhiya Al Buraidi

**Affiliations:** ^1^ Department of Internal Medicine, Nizwa Hospital, Nizwa, Oman; ^2^ Head, Neck and Thoracic program, Sultan Qaboos Comprehensive Cancer Care and Research Centre, University Medical City, Muscat, Oman; ^3^ Department of Pathology, Khoula Hospital, Muscat, Oman

## Abstract

Pulmonary lymphoma is a rare condition involving the lung characterized by the abnormal proliferation of lymphoid tissue. Patients may either be asymptomatic or exhibit nonspecific clinical presentations and radiologic findings, making diagnosis challenging. Here, we describe a case of a female patient who presented to our clinic with an incidental radiological finding of pulmonary consolidation/mass. The final diagnosis of a primary pulmonary marginal zone lymphoma required a tissue diagnosis.

## 1. Introduction

Primary pulmonary marginal zone lymphoma (PP‐MZL) is a rare subtype of non‐Hodgkin′s lymphoma (NHL), accounting for approximately 1%–2% of all primary pulmonary lymphomas [1, 2]. The presentation can be variable, with many cases being diagnosed incidentally on imaging. Symptomatic patients may exhibit nonspecific respiratory complaints such as cough, dyspnea, or recurrent infections. Radiographic findings range from solitary or multifocal pulmonary nodules to airspace consolidation and mass‐like lesions [3]. We present a case of a middle‐aged female who was incidentally found to have a mass in the right middle lobe during a computed tomography (CT) scan of the chest. This scan was performed as part of a general checkup abroad, sought for a second opinion due to her dissatisfaction with her overall health status. Histopathology of this mass confirmed the diagnosis of low‐grade B‐cell NHL consistent with marginal zone lymphoma.

## 2. Case Report

**Figure 2:**
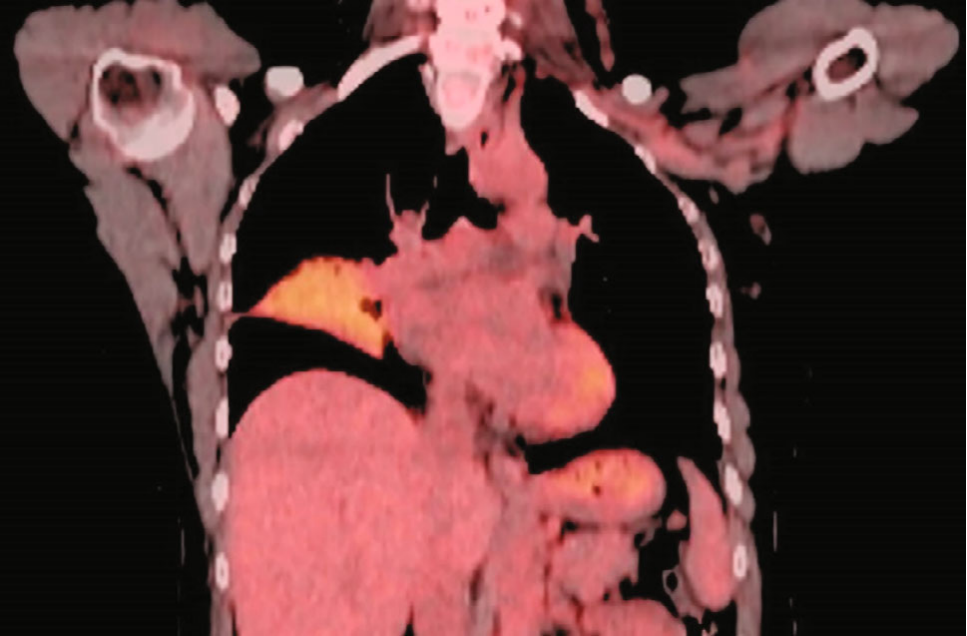
Positron emission tomography scan showing a diffusely avid consolidation (SUV max6.3) in the right middle lobe.

A 55‐year‐old female patient sought further evaluation at a secondary care hospital′s respiratory clinic due to an incidentally discovered lung mass. She has been suffering from alopecia areata and hair loss and has been following up with a dermatologist. She was unsatisfied with her medical progress and went abroad for a second opinion. There, she had multiple investigations, including a CT scan of the lung, which revealed a right middle lobe mass. Upon review at the respiratory clinic, she was free of respiratory symptoms and denied B symptoms like fever, sweating, and loss of weight. She is a lifetime nonsmoker and has no environmental or occupational exposures. Her medical examination was unremarkable. The blood investigations, including complete blood count (haemoglobin 12.8 g/dL, platelet count 224, white blood cells 3.9, neutrophils count 2.3, lymphocytes count 1, and eosinophils count.2), peripheral blood smear, renal function test, liver function test, LDH (187 IU/L), and bone profile were unremarkable. The repeated CT of the chest, abdomen, and pelvis showed a right middle lobe lateral segment and parahilar soft tissue density/consolidation with no obvious enhancement (Figure 1). The area measured 73∗32∗32 mm. The pulmonary veins and arteries were noted to pass through the mass and were patent. The CT abdomen showed multiple enlarged lymph nodes (portocaval and preceliac), gastric wall thickening, and a few small hepatic lesions that were difficult to characterize. The positron emission tomography (PET) scan showed diffusely avid right middle lobe consolidation (Figure 2), mildly avid epicardial, left internal mammary, retroperitoneal, mesenteric nodes, and differentially increased heterogenous metabolic activity in liver Segments II and III. The patient had a gastroscopy which showed severe gastritis, and multiple biopsies confirmed mild active chronic gastritis. The colonoscopy was unremarkable. Bronchoscopy revealed abnormally looking mucosa at the entrance of the lateral segment of the right middle lobe. Multiple endobronchial biopsies were obtained, and histopathology revealed respiratory epithelium with an underlying stroma that showed diffuse infiltration by sheets of medium‐sized lymphoid cells with a monocytoid appearance. They had small, irregular nuclei with small nucleoli and moderate cytoplasm. The immunohistochemistry of the lymphoid cells was diffusely positive for CD20, CD79a, PAX5, and BCL‐2. They were negative for CD5, CD10, BCL6, and cyclin D1. The Ki‐67 was low. These findings were in favour of small‐cell B cell NHL, favouring marginal zone lymphoma (Figure 3). Furthermore, the patient went through a CT‐guided biopsy which confirmed the same diagnosis. An endobronchial ultrasound‐guided transbronchial needle aspiration (EBUS‐TBNA) was also performed, but the cytopathology results were inconclusive. The patient was further evaluated by a clinical hematologist, who confirmed the impression of MZL stage IE and found no indication for treatment. The involved field radiation therapy (IFRT) was deferred due to the high risk of complications, given that the site is the lung. A year later, the patient developed anemia and work‐up revealed iron deficiency anemia (IDA). Her follow‐up PET CT showed progression of the right middle lobe lesion and new mild FDG uptake in abdominal and colonic areas. Therefore, a CT‐guided biopsy was done, and the histopathology was consistent with the previous diagnosis of extranodal marginal zone lymphoma (MZL) without any evidence of disease progression to a higher‐grade lymphoma. A repeated colonoscopy was unremarkable. Thereafter, the patient remained clinically stable and continued on a watch‐and‐wait strategy with ongoing close monitoring under the hematology team.

Figure 1Computed tomography of the chest coronal (a) and axial (b) views showing a consolidation with air bronchograms in the right middle lobe.

**Figure:**
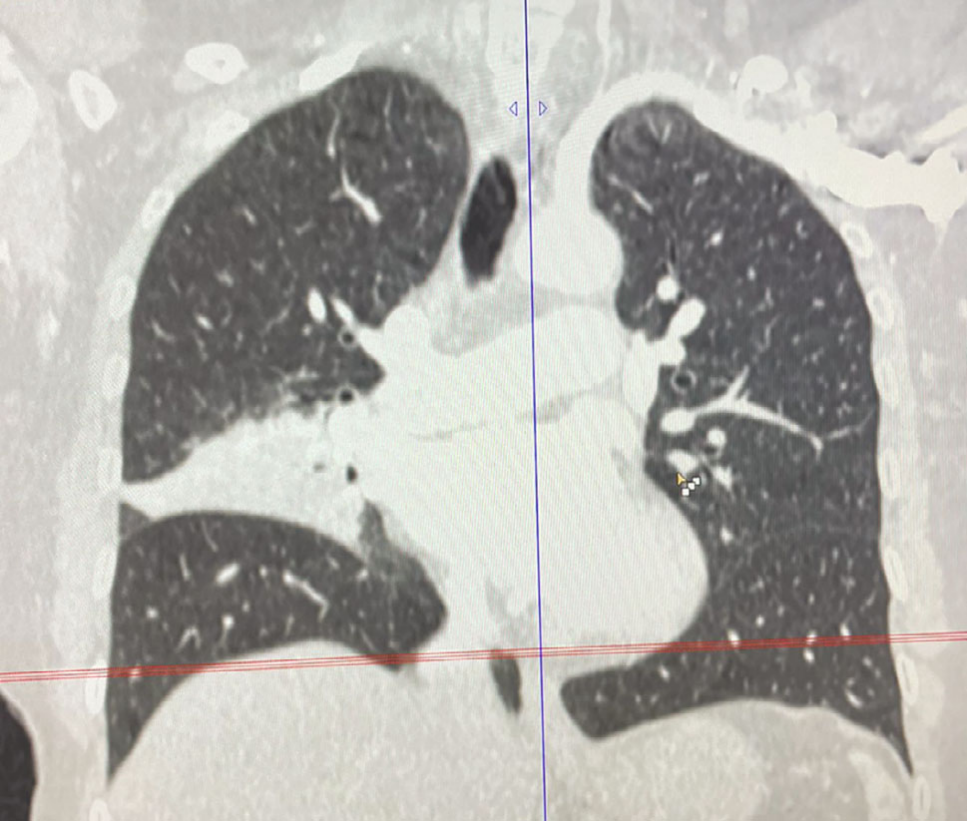
(a)

**Figure:**
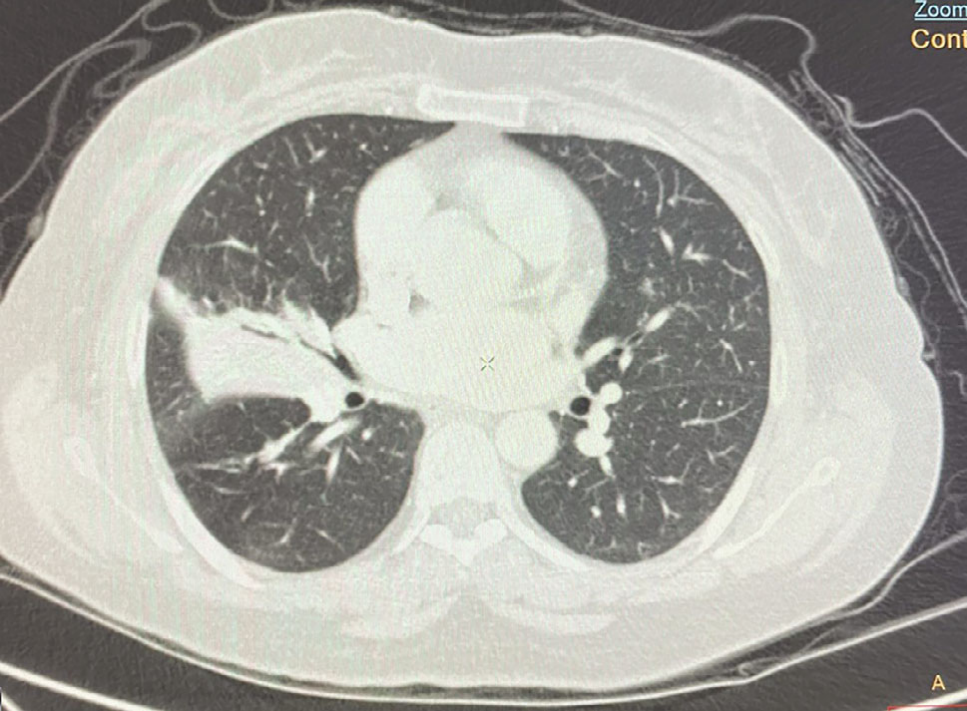
(b)

Figure 3(a) Section showing respiratory mucosa and the underlying stroma expanded by sheets of medium‐sized lymphocytes with monocytic appearance (H&E×10) and (b) PAX5 immunostaining highlights malignant lymphoid B cells.

**Figure:**
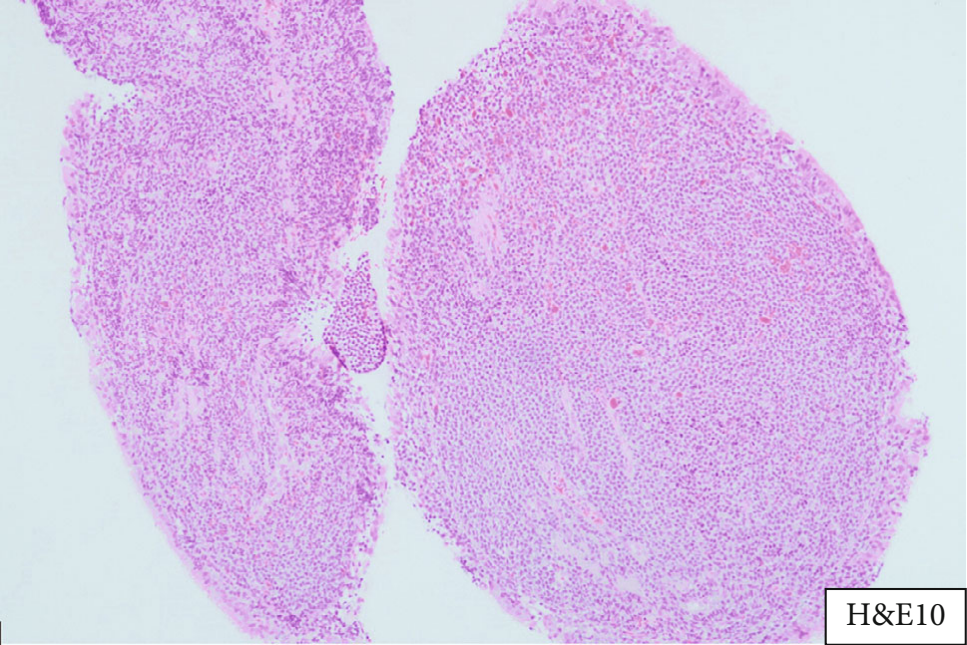
(a)

**Figure:**
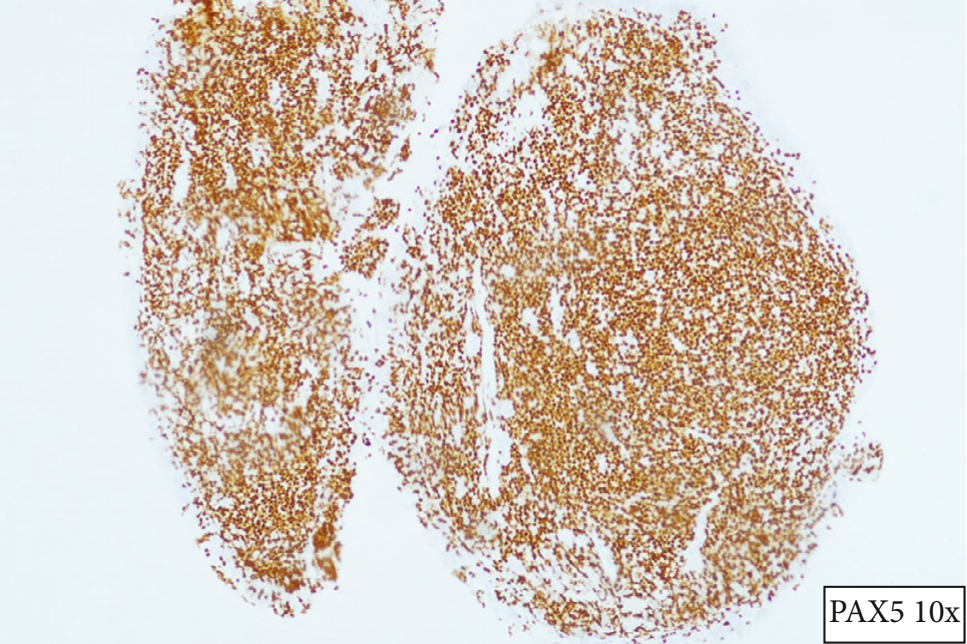
(b)

## 3. Discussion

Primary pulmonary non‐Hodgkin′s lymphoma is rare, with an incidence of approximately 0.3% among primary lung malignancies [4]. PP‐MZL, a form of mucosa‐associated lymphoid tissue (MALT) lymphoma, comprises 9%–14% of MALT lymphomas [5]. It is predominantly diagnosed in females, with a median age of 67 years. More than 80% of cases present at Stage I or II [6]. PP‐MZL arises from bronchial‐associated lymphoid tissue (BALT), which may develop due to chronic inflammation, smoking, or autoimmune conditions [7]. Smoking has been implicated in about one‐third of cases [8].

Chronic antigenic stimulation has been proposed as an important etiologic mechanism. Similar to the well‐established association between *Helicobacter pylori* and gastric MALT lymphoma, infection‐driven lymphoproliferation has been suggested in pulmonary MALT lymphoma [9]. Reported microbial triggers include *Chlamydia psittaci*, *Achromobacter xylosoxidans*, and *Mycoplasma* species, although a definitive causal organism has not been confirmed. Our patient′s history of dyspeptic symptoms and coexistent gastritis makes this infectious association clinically relevant and supports the concept that chronic antigenic stimulation, whether from respiratory or gastrointestinal sources, may contribute to lymphomagenesis. This highlights the importance of investigating infectious and autoimmune comorbidities in patients presenting with pulmonary MALT lymphoma [10].

Most patients are asymptomatic, with incidental radiographic findings [11]. Symptoms, when present, are nonspecific (cough, mild dyspnea, and chest pain) and do not correlate well with disease severity [12]. Our patient, notably, was asymptomatic despite radiological abnormality, aligning with published data indicating that approximately one‐third of cases are discovered incidentally [9]. This underscores the diagnostic challenge of differentiating PP‐MZL from other causes of nonresolving pulmonary consolidation such as infection or organizing pneumonia findings, emphasizing the diagnostic challenges.

Radiologically, PP‐MZL may mimic infectious consolidation, nodular masses, or ground‐glass opacities [10]. Tissue biopsy remains the gold standard for diagnosis, often requiring bronchoscopy or CT‐guided biopsy. Immunophenotypic analysis is crucial, with PP‐MZL cells typically expressing CD20, CD19, CD22, and CD79a, and negative for CD5 and cyclin D1 [13]. The t (11;18) translocation is found in about 40% of cases [14].

The clinical course of PP‐MZL is heterogeneous. While many cases follow an indolent, self‐limiting trajectory, others show slow but progressive pulmonary or extrapulmonary involvement requiring systemic therapy. Kou et al. [10] reported an asymptomatic patient with persistent consolidation that remained radiologically stable for months before definitive diagnosis, emphasizing that nonresolving lesions should prompt consideration of lymphoma even in the absence of symptoms. Conversely, Wang et al. [9] analyzed 80 patients and demonstrated that 16% developed progression or relapse, with age and tumor burden (number of lesions) influencing survival outcomes. Their findings highlight that while “watch‐and‐wait” is acceptable for localized, asymptomatic disease, close surveillance is essential, as a subset may evolve to require chemotherapy or immunotherapy.

Our patient was managed conservatively after diagnosis, with regular clinical and radiological follow‐up. Over a 24‐month period, the lesion remained stable in size and appearance, and no systemic manifestations developed. This observation supports the indolent nature of PP‐MZL in selected patients and aligns with the long‐term stability reported in untreated cases by Troch et al. [15] and Borie et al. [16]. Nonetheless, multidisciplinary evaluation remains crucial to balance observation against the risk of transformation to higher‐grade lymphoma.

Prognosis is generally excellent, with 5‐ and 10‐year disease‐specific survival rates estimated at 90% and 70%, respectively [17]. Wang et al. [9] reported a 5‐year overall survival of 87%, confirming the favourable outcome across various treatment strategies. Therapeutic approaches include watchful waiting, localized radiotherapy, surgery, or systemic immunochemotherapy [18]. Management should be individualized according to stage, comorbidities, and disease kinetics, with rituximab‐based regimens reserved for disseminated or symptomatic cases.

## 4. Conclusion

In summary, we present a case of primary pulmonary marginal zone lymphoma, which was particularly challenging given that the patient was asymptomatic. The differential diagnosis of the radiological findings in this case is broad, which significantly increases the difficulty of the clinical diagnosis.

## Conflicts of Interest

The authors declare no conflicts of interest.

## Funding

No funding was received for this manuscript.

## Data Availability

The data that support the findings of this study are available from the corresponding author upon reasonable request.

## References

[1] Cadranel J. , Wislez M. , and Antoine M. , Primary Pulmonary Lymphoma, European Respiratory Journal. (2002) 20, no. 3, 750–762, 10.1183/09031936.02.00404102, 2-s2.0-0036738162. 12358356

[2] Sanguedolce F. , Zanelli M. , Zizzo M. , Bisagni A. , Soriano A. , Cocco G. , Palicelli A. , Santandrea G. , Caprera C. , Corsi M. , Cerrone G. , Sciaccotta R. , Martino G. , Ricci L. , Sollitto F. , Loizzi D. , and Ascani S. , Primary Pulmonary B-Cell Lymphoma: A Review and Update, Cancers (Basel). (2021) 13, no. 3, 10.3390/cancers13030415, 33499258. PMC7865219 33499258

[3] Borie R. , Wislez M. , Antoine M. , and Cadranel J. , Lymphoproliferative Disorders of the Lung, Respiration. (2017) 94, no. 2, 157–175, 10.1159/000477740, 2-s2.0-85024397833. 28609772

[4] Ferraro P. , Trastek V. F. , Adlakha H. , Deschamps C. , Allen M. S. , and Pairolero P. C. , Primary Non-Hodgkin′s Lymphoma of the Lung, Annals of Thoracic Surgery. (2000) 69, no. 4, 993–997, 10.1016/S0003-4975(99)01535-0, 2-s2.0-0034057957. 10800781

[5] Cheah C. Y. and Seymour J. F. , Marginal Zone Lymphoma: 2023 Update on Diagnosis and Management, American Journal of Hematology. (2023) 98, no. 10, 1645–1657, 10.1002/ajh.27058. 37605344

[6] Sammassimo S. , Pruneri G. , Andreola G. , Montoro J. , Steffanoni S. , Nowakowski G. S. , Gandini S. , Negri M. , Habermann T. M. , Raderer M. , Li Z. M. , Zinzani P. L. , Adam P. , Zucca E. , and Martinelli G. , A Retrospective International Study on Primary Extranodal Marginal Zone Lymphoma of the Lung (BALT Lymphoma) on Behalf of International Extranodal Lymphoma Study Group (IELSG), Hematological Oncology. (2016) 34, no. 4, 177–183, 10.1002/hon.2243, 2-s2.0-84936806460. 26152851

[7] Kelemen K. , Rimsza L. M. , and Craig F. E. , Primary Pulmonary B-Cell Lymphoma, Seminars in Diagnostic Pathology. (2020) 37, no. 6, 259–267, 10.1053/j.semdp.2020.04.002. 32444246

[8] Zucca E. and Bertoni F. , The Spectrum of MALT Lymphoma at Different Sites: Biological and Therapeutic Relevance, Blood. (2016) 127, no. 17, 2082–2092, 10.1182/blood-2015-12-624304, 2-s2.0-84993997752, 26989205. 26989205

[9] Wang L. , Ye G. , Liu Z. , Shi L. , Zhan C. , Gu J. , Luo R. , Lin Z. , Ge D. , and Wang Q. , Clinical Characteristics, Diagnosis, Treatment, and Prognostic Factors of Pulmonary Mucosa-Associated Lymphoid Tissue-Derived Lymphoma, Cancer Medicine. (2019) 8, no. 18, 7660–7668, 10.1002/cam4.2683, 31691549. 31691549 PMC6912039

[10] Kou L. , Huan N. C. , Nyanti L. E. , Chin J. S. , Mohamad N. B. , and Ramarmuty H. Y. , Pulmonary Extra-Nodal Mucosa-Associated Lymphoid Tissue (MALT) Lymphoma: A Rare Cause of Persistent Lung Consolidation, Respirol Case Rep.(2023) 11, no. 8, e01197, 10.1002/rcr2.1197, 37501686. 37501686 PMC10368649

[11] O′Donnell P. G. , Ahmad F. , Flower C. D. R. , McCafferty I. , and Shorvon P. J. , Radiological Appearances of Lymphomas Arising From mucosa-associated lymphoid tissue (MALT) in the Lung, Clinical Radiology. (1998) 53, no. 4, 258–263, 9585040. 9585040 10.1016/s0009-9260(98)80123-2

[12] Bi W. , Chen D. , She Y. , Zhang L. , Yang C. , Zhang J. , Yin W. , Liang W. , and He J. , Pulmonary Mucosa-Associated Lymphoid Tissue Lymphoma: CT Findings and Pathological Basis, Journal of Surgical Oncology. (2021) 123, no. 5, 1336–1344, 10.1002/jso.26403, 33523526. 33523526

[13] Husnain M. , Kuker R. , Reis I. M. , Iyer S. G. , Zhao W. , Chapman J. R. , Vega F. , Lossos I. S. , and Alderuccio J. P. , Clinical and Radiological Characteristics of Patients With Pulmonary Marginal Zone Lymphoma: A Single-Center Analysis, Cancer Medicine. (2020) 9, no. 14, 5051–5064, 10.1002/cam4.3096, 32452658. 32452658 PMC7367627

[14] Tang V. K. , Vijhani P. , Cherian S. V. , Ambelil M. , and Estrada-Y-Martin R. M. , Primary Pulmonary Lymphoproliferative Neoplasms, Lung India. (2018) 35, no. 3, 220–230, 10.4103/lungindia.lungindia_381_17, 2-s2.0-85048368349, 29697079. 29697079 PMC5946555

[15] Troch M. , Streubel B. , Petkov V. , Turetschek K. , Chott A. , and Raderer M. , Does Malt Lymphoma of the Lung Require Immediate Treatment? An Analysis of 11 Untreated Cases with Long-term Follow-Up, Anticancer Research. (2007) 27, no. 5B, 3633–3637, 17972528. 17972528

[16] Borie R. , Wislez M. , Thabut G. , Antoine M. , Rabbat A. , Couderc L. J. , Monnet I. , Nunes H. , Blanc F. X. , Mal H. , Bergeron A. , Dusser D. , Israël-Biet D. , Crestani B. , and Cadranel J. , Clinical Characteristics and Prognostic Factors of Pulmonary MALT Lymphoma, European Respiratory Journal. (2009) 34, no. 6, 1408–1416, 10.1183/09031936.00039309, 2-s2.0-73249151812. 19541720

[17] Kurtin P. J. , Myers J. L. , Adlakha H. , Strickler J. G. , Lohse C. , Pankratz V. S. , and Inwards D. J. , Pathologic and Clinical Features of Primary Pulmonary Extranodal Marginal Zone B-Cell Lymphoma of MALT Type, American Journal of Surgical Pathology. (2001) 25, no. 8, 997–1008, 10.1097/00000478-200108000-00003, 2-s2.0-0034933899, 11474283. 11474283

[18] Kaddu-Mulindwa D. , Thurner L. , Christofyllakis K. , Bewarder M. , and Kos I. A. , Management of Extranodal Marginal Zone Lymphoma: Present and Upcoming Perspectives, Cancers (Basel). (2022) 14, no. 12, 10.3390/cancers14123019, 35740684. PMC9220961 35740684

